# Survivors of war in northern Kosovo (III): The role of anger and hatred in pain and PTSD and their interactive effects on career outcome, quality of sleep and suicide ideation

**DOI:** 10.1186/1752-1505-6-4

**Published:** 2012-07-30

**Authors:** Shr-Jie Wang, Feride Rushiti, Xhevdet Sejdiu, Sebahate Pacolli, Besart Gashi, Florentina Salihu, Jens Modvig

**Affiliations:** 1 Rehabilitation and Research Centre for Torture Victims (RCT), Copenhagen, Denmark; 2 Kosova Rehabilitation Centre for Torture Victims (KRCT), Pristina, Kosovo; 3 Department of Psychology, University of Pristina, Pristina, Kosovo; 4 Faculty of Medicine, University of Pristina, Pristina, Kosovo

## Abstract

**Background:**

The management of chronic debilitating health conditions after trauma remains a challenge in post-conflict settings. The study aimed to expand current understanding of the diagnostic overlap of pain and PTSD and explore their independent and interactive effect on career change, sleep disorder and suicide ideation. The role of anger and hatred as contributing factors to the persistence of pain and PTSD were also examined.

**Methods:**

125 victims of torture and massive violence identified in a household survey took part in the in-depth assessment. Socio-demographic data and information on trauma, emotional disturbance, injuries and medication history were collected. PTSD was diagnosed according to DSM-IV criteria. Pain was assessed using the McGill Pain Questionnaire and the Margolis Pain Diagram.

**Results:**

Nearly 95% participants experienced pain during the last 2 weeks, 47% were diagnosed with PTSD, 50% were taking medication against depression and anxiety. There is substantial overlap of pain, PTSD and emotional disturbance. Injury history, PTSD and negative emotions were related to the pain score and the number of pain locations. Anger, hatred or an inferiority complex particularly amplified pain experience. Headache was constant and especially prevalent in those with recent experience of anger, aggressiveness and hatred. The risk of having chest and abdominal pain within 2 weeks was very high in those who had chest injury and had recently been crying. An increased risk of changing jobs or stopping work or schooling due to depression or injury was observed for those with a higher pain score, and for pain in neck, shoulder and upper limbs. The prevalence of sleep disorders was 80%, that of suicide ideation 70%, and these were found to be associated with greater pain and anger. PTSD was also related to suicide ideation.

**Conclusions:**

The findings provide an overview of pain characteristics in individuals with PTSD and injury and confirm the hypothesized effects on career outcome, sleep disorders and suicide ideation. The study revealed a high level of persistent anger and hatred. The findings suggest the need for new approaches to rehabilitation in a post-war setting, including ways in which to address collective emotional hurt in the society.

## Background

There is mounting evidence that, many years after the encounter with torture and massive violence, many survivors suffer not only from post-trauma stress disorder (PTSD), but also from diverse somatic symptoms including chronic pain.

Numerous studies have examined the comorbidity of pain and PTSD in the general population or among war veterans and the impact of this on quality of life [1-5]. Chronic pain often occurs together with PTSD and adversely affects the response of these conditions to psychiatric treatment [6]. It may lead to more severe depression or pain-related functional disability. It is often correlated with a high unemployment rate and more frequent use of medication and health care services [7].

Over the last two decades there has been a growing appreciation of the long-term effects of trauma resulting from torture and massive violence, and pain has been found to be the most common complaint among torture victims [8-10]. Pain could be one of the most common complaints among the general population in fragile and conflict-affected countries too. In a population-based study of violence and human rights violations in Mitrovicë district of Kosovo [11], we observed a high prevalence of pain complaints (both mental and physical). The proportion of family members with pain complaints was related to the level of violence and human rights violations, to which the household had been exposed, and also to a decline in household income due to the presence of an injured person.

We have also performed a subsequent detailed investigation of a group of victims of torture and massive violence identified in the population-based study. One aim of the detailed investigation was to add to our knowledge of comorbidity of pain and PTSD among the victims. We hope that more detailed information about debilitating health conditions in a post-conflict setting can be used to support the development of effective rehabilitation and peace-building approaches for a traumatized population. A subsidiary aim was to show that the standardized pain assessment tools that we used, McGill Pain Questionnaire (MPQ) [12,13] and the Margolis Pain Diagram [14], could be applied in routine health screening in a post-war setting such as that of Kosovo.

In the present paper we focus on the characteristics of the pain experienced by individuals with PTSD, emotional disturbance or injury, and also examine their independent and interactive effects on three outcomes of interest. First, we examined the difference in pain characteristics (sensory and affective dimension of pain) and the pain rating index (PRI) of the MPQ in a group of victims with PTSD, emotional disturbance or bodily injuries. We further examined the association of particular pain locations and the total number of pain locations with PTSD, emotional disturbance and injury, and finally, we investigated their interactive effect on outcomes such as changing jobs or no longer attending school, sleep disorders and suicide ideation. We hypothesized that such outcomes would be more frequent in people with comorbidity of emotional, mental and/or physical problems.

## Methods

### Study design and implementation

The study reported here is part of a large population-based study, carried out in 2008 in three of six municipalities in Mitrovicë district: Mitrovicë, Skënderaj and Vushtrri municipalities where Albanians dominate. The general methodology of this study, including the processes of participant selection, inclusion and exclusion criteria and data collection have been described in detail in previous publications [11,15] and will only be summarized here.

We recruited a group of 125 victims from the households that reported one of the following experiences during the household survey: 1) torture and other cruel, inhuman or degrading treatment or punishment; 2) sexual harassment, molestation, rape or insertion of a blunt object into a genital organ and/or the rectum; 3) arrest and detention without warrant or order; or 4) extrajudicial execution of family members, perpetrated by members of a law enforcement agency. The definition of torture strictly follows the United Nations Convention Against Torture Article 1.1 [16]. The 125 people took part in a detailed interview and clinical and functional assessment at three mobile clinics.

The extensive interviews with all participants were conducted by ten experienced interviewers and one senior clinical psychologist. They were all Kosovar Albanians except for one Kosovar Turk. They used a set of standard questionnaires. Some parts were translated into Albanian by professional translators and validated by standard translation-retranslation and some parts were adapted from the questions that have been validated in the same population in the previous studies [17-19]. Demographic data and data on torture/abuse experience were collected using the guideline of the Istanbul Protocol: the UN manual on the effective investigation and documentation of torture and other cruel, inhuman or degrading treatment or punishment [20]. Experiences of emotional disturbance such as feelings of anger or hatred*,* aggressiveness, sleep disorder and military or police phobia within the two weeks before the interview were self-reported. Suicide ideation was assessed using the following question: “Over the last 2 weeks, how often have you been bothered by thoughts that you would be better off dead or of hurting yourself in some way?”

The participants also underwent clinical and functional assessments by three medical doctors and a physiotherapist who were all Kosovar Albanians and had received training in trauma care. XS is a principal neuropsychiatric specialist; FR and SP have extensive experience in collecting testimony and providing trauma care. A PTSD diagnosis was established in those cases meeting the diagnostic criteria in the American Psychiatric Association’s Diagnostic and Statistical Manual of Mental Disorders (DSM-IV) and the findings were interpreted in the local context.

Injuries were examined by medical doctors, were recorded using body diagrams to mark the location and were counted subsequently. Detailed results have been published elsewhere [15].

Baseline pain level was assessed by medical doctors using a short form of MPQ which has been tested widely and found to be an instrument capable of providing information about both sensory and affective dimensions of pain [12,13]. The questionnaire lists 11 sensory and five affective descriptors, which provide a measurement of the pain itself (sensory component) and of the unpleasant feelings and emotions resulting from the pain experience (affective component). Participants are asked to rate the intensity of each descriptor of pain as follows: 0 “no”, 1 “mild”, 2 “moderate” or 3 “severe pain”. Scores are summed from the values attributed to the words chosen for sensory and for affective descriptors. A pain rating index (PRI) is then calculated as the total of the values summed from the score of 16 descriptors [12,21,22]. Location of pain was self-reported and recorded on a drawing outlining a male or a female figure: the Margolis Pain Diagram [14].

### Statistical analysis

Data were entered and validated in Microsoft Access 2000. Cross-checking was done for quality control. Discrepancies between the original paper forms and the dataset were corrected: one mismatched record was eliminated. The data was analysed using the Stata statistical package, version 11.0 (StataCorp LP, Texas, USA, 2009). Frequency distributions and cross-tabulations were generated to compare the individual variables. Bivariate analysis for differences in means was carried out using logistic and linear regression models, adjusted for the effects of confounding factors. The outcomes of interest are pain characteristics (sensory and affective dimensions of pain and PRI), career outcome (changing jobs, stopping work or stopping schooling due to depression or injury), self-reported sleep disorders and suicide ideation. Confounders such as sex, marital status, education, income level, geographical location of residence and having taken medication against anxiety and depression were adjusted for in the analysis. An alpha level of P = 0.05 was set to denote the significance of analysis. All P values are expressed to 2 digits to the right of the decimal point, unless P <0.01, in which case the P value is expressed to, at most, 3 digits.

### Ethical evaluation

The study conformed with Danish law and adhered to the Declaration of Helsinki. The Ethical Committee of the Academy of Medical Sciences of Kosovo approved the study. The participants were informed of the objectives and procedure of the study and the use of the data, as well as being assured of anonymity and the confidential treatment of information they provided. The participants all provided verbal consent. Further diagnosis and brief counselling for participants were provided in simple cases of torture or abuse, while severe cases were referred to the municipal family health centres where a group of medical doctors had previously been trained by KRCT to take care of traumatised people.

## Results

### Socio-demographic profile of victims of massive violence

The mean age of the 63 male and 62 female participants was approximately 47 years. A detailed demographic profile was published previously [15]. All of them were Albanians and 52 (41.6% were affiliated with the Kosovo Liberation Army (KLA). Overall, 32.8% were unemployed, 24% were pensioners and 32% (including two males) classified themselves as household workers.

**Figure 1:**
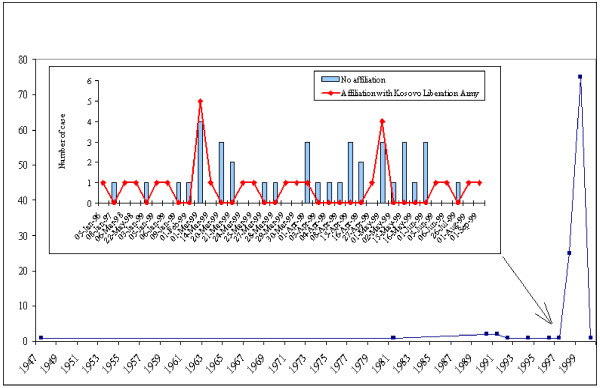
Trend and patterns of incident reporting.

Twenty-eight (22.4%) had been in a combat or cross-fire situation, 88 (70.4%) had witnessed a family member or a relative being arrested, assaulted, humiliated, tortured or killed, 35 (28.0%) had been arrested or detained without a warrant, 101 (80.1%) had been subjected to torture and other cruel, inhuman or degrading treatment or punishment, 12 (9.6%) had been forced into labour. A few traumatic events had occurred in 1947 and in 1981 during Tito's rule in Yugoslavia (1945–1986) (Figure 1). The majority of cases had occurred in 1997–1998 and after the start of the NATO bombing campaign in March 1999. Five participants had been hit, slapped, kicked or otherwise physically hurt or had been forced into an unwanted sexual act by someone within the 12 months preceding the survey in 2007–2008. The events most frequently took place at their places of residence (38.3%), on the street or motorway (28.0%) or in the wild (16.0%). Members of the Serbian paramilitary forces (40.7%), the Serbian military police (26.2%) and the police (18.6%) were the major perpetrators, and they were also involved in the two cases of sexual assault. Gangsters were responsible for a few of the violent acts too. Twenty-seven participants (21.6%) had pursued legal action against the perpetrators and 23 of them (18.4%) reported that the perpetrators had been prosecuted.

### PTSD diagnoses and symptom burden

Fifty-nine out of 125 participants (47%) met diagnostic criteria for PTSD, 100 (80%) reported sleep disorder and 82 (75%) had had suicide thoughts within 2 weeks preceding the study. Among those with PTSD, 35 had taken medication against depression or anxiety, 45 reported military or police phobia and within 2 weeks preceding the study, 50 had suicide ideation: 16 once in several days, 27 nearly half of the days and 7 almost every day, 52 reported sleep disorders and 57 had experienced anger.

### Pain and its association with injury and other variables

There were no strong associations between pain frequency and the following variables: PTSD, the number of bodily injuries or most of the emotional disturbances. Ninety-one out of 125 participants (74%) showed at least one bodily injury on the body map and 95% experienced pain. In an unadjusted model, associations with the number of bodily injuries were consistent with a pain threshold rather than with a linear effect; that is, there was a significant difference in pain score between the participants with any number of bodily injuries and those without any injury. Table 1 presents the composite scores for pain assessment from the MPQ. Higher sensory dimensional scores were reported in participants with PTSD or with one or more bodily injuries, although the latter effect is attenuated by model adjustment. Participants with one or more bodily injury showed significantly higher score on the affective dimension and PRI. The participants with PTSD did not show higher score on the affective dimension than those without PTSD; and the association between PTSD and PRI was attenuated by adjustment. Increase in all the pain scores was associated with most of the emotional disturbances and the scores were significantly higher in participants reporting hatred, inferiority complexes and helplessness as well as military or police phobia (Table 1). In particular, experiencing anger within two weeks preceding the survey appeared to be the strongest predictor of PRI (OR = 11.81, 95% CI: 6.61-17.01, P < 0.001).

**Table 1:** Pain characteristics and pain rating index compared by mental, physical and emotional problems

	**Score of sensory component**			**Score of affective component**			**Pain rating index**		
**Mental and physical problems**	**N**	**Unadjusted OR (95% CI) P value**	Multiply adjusted*	**N**	**Unadjusted OR (95% CI) P value**	Multiply adjusted*	**N**	**Unadjusted OR (95% CI) P value**	Multiply adjusted*
PTSD	107	2.91 (0.99, 4.82)	2.13 (0.32, 3.94)	111	0.42 (−1.10, 1.93)	−0.23 (−1.59, 1.12)	107	3.35 (0.39, 6.32)	1.87 (−0.73, 4.48)
		*P < 0.01*	*P < 0.05*		*P = 0.59*	*P = 0.74*		*P < 0.05*	*P = 0.16*
Number of injuries	117	2.80 (0.58, 5.01) *P < 0.01*	0.61 (−1.74, 2.96) *P = 0.61*	121	5.06 (3.55, 6.56) *P < 0.001*	3.93 (2.39, 5.47) *P < 0.001*	117	7.96 (4.71, 11.20) *P < 0.001*	4.56 (1.21, 7.92) *P < 0.01*
0 vs. ≥1 injuries									
**Emotional disturbance**	**N (no/yes)**	**Unadjusted OR (95% CI) P value**	Multiply adjusted*	**N (no/yes)**	**Unadjusted OR (95% CI) P value**	Multiply adjusted*	**N (no/yes)**	**Unadjusted OR (95% CI) P value**	Multiply adjusted*
Anger	9/108	9.34 (5.95, 12.73)	7.34 (3.76, 10.93)	10/111	5.85 (3.23, 8.46)	3.88 (1.27, 6.48)	9/108	15.76 (10.57, 20.95)	11.81 (6.61, 17.01)
		*P < 0.001*	*P < 0.001*		*P < 0.001*	*P < 0.01*		*P < 0.001*	*P < 0.001*
Aggressiveness	40/77	4.06 (2.06, 6.06)	2.38 (0.23, 4.53)	42/79	3.23 (1.71, 4.75)	1.83 (0.29, 3.37)	40/77	7.50 (4.46, 10.54)	4.39 (1.27, 7.52)
		*P < 0.001*	*P < 0.05*		*P < 0.001*	*P < 0.05*		*P < 0.001*	*P < 0.01*
Crying	33/84	0.97 (−1.28, 3.21)	1.19 (−1.05, 3.43)	35/86	2.12 (0.45, 3.79)	2.28 (0.71, 3.85)	33/84	3.22 (−0.25, 6.70)	3.60 (0.35, 6.84)
		*P = 0.40*	*P = 0.30*		*P < 0.01*	*P < 0.01*		*P = 0.07*	*P < 0.05*
Hatred	44/73	2.19 (0.14, 4.25)	2.33 (0.34, 4.32)	47/74	2.41 (0.88, 3.95)	2.46 (1.06, 3.86)	44/73	4.74 (1.59, 7.90)	4.96 (2.11, 7.80)
		*P < 0.05*	*P < 0.05*		*P < 0.01*	*P < 0.001*		*P < 0.01*	*P < 0.001*
Helplessness	33/84	2.19 (−0.03, 4.41)	2.19 (0.06, 4.31)	34/87	2.46 (0.79, 4.13)	2.49 (1.01, 3.98)	33/84	4.83 (1.42, 8.24)	4.78 (1.73, 7.83)
		*P < 0.05*	*P < 0.05*		*P < 0.01*	*P < 0.001*		*P < 0.01*	*P < 0.01*
Hopelessness	35/82	2.19 (0.01, 4.36)	1.13 (−1.05, 3.31)	38/83	3.39 (1.83, 4.95)	2.44 (0.93, 3.94)	35/82	5.72 (2.42, 9.02)	3.71 (0.56, 6.86)
		*P < 0.05*	*P = 0.31*		*P < 0.001*	*P < 0.01*		*P < 0.001*	*P < 0.05*
Inferiority complex	41/76	3.26 (1.22, 5.29)	2.28 (0.22, 4.33)	42/79	3.89 (2.42, 5.36)	2.95 (1.54, 4.36)	41/76	7.35 (4.32, 10.39)	5.33 (2.42, 8.25)
		*P < 0.01*	*P < 0.05*		*P < 0.001*	*P < 0.001*		*P < 0.001*	*P < 0.001*
Military or police phobia	35/82	3.16 (1.03, 5.30)	1.61 (−0.74, 3.97)	37/84	4.39 (2.91, 5.88)	3.01 (1.40, 4.61)	35/82	7.76 (4.60, 10.91)	4.78 (1.41, 8.15)
		*P < 0.01*	*P = 0.18*		*P < 0.001*	*P < 0.001*		*P < 0.001*	*P < 0.01*
Sadness	48/69	1.01 (−1.04, 3.06)	1.52 (−0.54, 3.58)	50/71	0.28 (−1.30, 1.85)	0.59 (−0.91, 2.09)	48/69	1.34 (−1.88, 4.56)	2.14 (−0.89, 5.17)
		*P = 0.33*	*P = 0.15*		*P = 0.73*	*P = 0.44*		*P = 0.41*	*P = 0.17*

*Adjusted for sex, marital status, education, income, municipality and medication.

On the Margolis Pain Diagram, 52.0% of participants (n = 65) indicated 1–3 locations, 32.8% had 4–6 locations and 6.4% had 7–10 locations. Adjusting for sex, marital status, education, income, municipality and medication in the model, we again observed an association between pain and anger; reporting of more pain locations was most marked in participants who reported experiencing anger in the previous 2 weeks (OR = 2.51, 95% CI: 1.19-3.83, P < 0.001).

**Figure 2:**
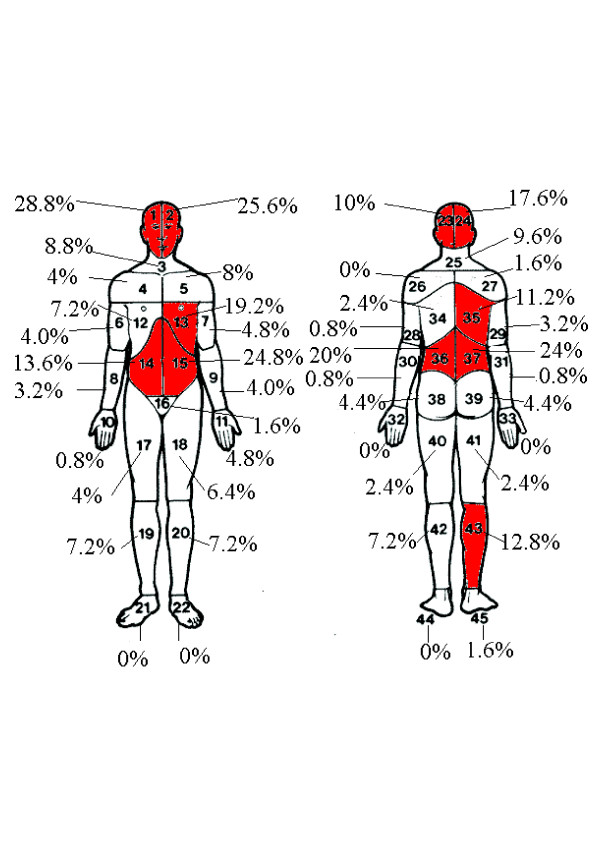
Pain locations on body diagram.

The most common pain location was the front of the head or the lower back (Figure 2). Pain locations on the Margolis Pain Diagram were combined as (a) head (sites 1, 2, 23, 24); (b) neck and shoulder (3, 4, 5, 25, 26, 27); (c) chest and abdomen (12, 13, 14, 15, 16); (d) back (34, 35, 36, 37, 38, 39); (e) upper limbs (6–11, 28–33); and (f) lower limbs (17–22, 40–45) for further data analysis. Table 2 lists the significant variables for different pain locations. Non-specific headache was somewhat prevalent in those with PTSD (the association was attenuated), but more markedly associated with recent experience of anger, aggressiveness and hatred (Table 2). Significant increase in chest and abdominal pain was observed in those having one or more injuries (OR = 8.18, 95% CI = 2.53-26.41, P < 0.001) and who reported crying in the previous two weeks (OR = 13.15, 95% CI: 3.82-45.25, P < 0.001). Chest and abdominal pain was also associated with anger, but this result was based on the very small number of participants who did not report anger (n = 11) and was therefore substantially attenuated by adjustment. Back pain was markedly more prevalent in those who reported feeling hatred while its association with having one or more injuries was attenuated by adjustment. The participants with a PTSD diagnosis tended to report more upper and lower limb pain (Table 2).

**Table 2:** Odds ratios for different pain locations by mental and physical problems and selected emotional disturbances

**Pain located in the head or in neck and shoulders**				
**Head**	**N (no/yes)**	**Unadjusted OR (95% CI) P value**	Multiply adjusted for confounders*	Additionally adjusted for other pain locations**
PTSD	34/20	1.00	1.00	1.00
	31/27	1.48 (0.70, 3.15)	1.56 (0.69, 3.52)	1.79 (0.75, 4.26)
		*P = 0.31*	*P = 0.28*	*P = 0.19*
Number of injuries	18/16	1.00	1.00	1.00
	55/34	0.70 (0.31, 1.54)	0.65 (0.26, 1.62)	0.66 (0.24, 1.82)
		*P = 0.37*	*P = 0.35*	*P = 0.42*
Anger in last 2 weeks	8/3	1.00	1.00	1.00
	65/47	1.93 (0.49, 7.66)	2.47 (0.55, 11.20)	5.24 (1.01, 27.28)
		*P = 0.35*	*P = 0.24*	*P < 0.05*
Aggressiveness in last 2 weeks	32/11	1.00	1.00	1.00
	41/39	2.77 (1.23, 6.24)	3.32 (1.30, 8.49)	3.64 (1.38, 9.59)
		*P < 0.01*	*P < 0.01*	*P < 0.01*
Crying in last 2 weeks	24/13	1.00	1.00	1.00
	50/38	1.31 (0.59, 2.92)	1.35 (0.54, 3.37)	1.72 (0.62, 4.83)
		*P = 0.51*	*P = 0.52*	*P = 0.30*
Hatred in last 2 weeks	33/15	1.00	1.00	1.00
	40/35	1.93 (0.90, 4.12)	1.61 (0.71, 3.66)	2.19 (0.91, 5.26)
		*P = 0.09*	*P = 0.25*	*P = 0.08*
**Neck and shoulder pain**	**N (no/yes)**	**Unadjusted OR (95% CI) P value**	Multiply adjusted for confounders*	Additionally adjusted for other pain locations**
PTSD	42/12	1.00	1.00	1.00
	39/19	1.71 (0.73, 3.97)	1.52 (0.62, 3.74)	1.50 (0.58, 3.88)
		*P = 0.22*	*P = 0.37*	*P = 0.40*
Number of injuries	27/7	1.00	1.00	1.00
	63/26	1.59 (0.62, 4.11)	1.35 (0.47, 3.85)	1.47 (0.45, 4.86)
		*P = 0.34*	*P = 0.58*	*P = 0.53*
Anger in last 2 weeks	10/1	1.00	1.00	1.00
	80/32	4.00 (0.49, 32.54)	4.37 (0.46, 41.34)	2.93 (0.26, 33.18)
		*P = 0.20*	*P = 0.20*	*P = 0.39*
Aggressiveness in last 2 weeks	34/9	1.00	1.00	1.00
	56/24	1.62 (0.67, 3.89)	1.50 (0.57, 3.95)	1.52 (0.53, 4.34)
		*P = 0.28*	*P = 0.41*	*P = 0.43*
Crying in last 2 weeks	29/8	1.00	1.00	1.00
	63/25	1.41 (0.57, 3.52)	1.97 (0.71, 5.49)	2.39 (0.74, 7.77)
		*P = 0.46*	*P = 0.19*	*P = 0.15*
Hatred in last 2 weeks	33/15	1.00	1.00	1.00
	57/18	0.69 (0.31, 1.56)	0.82 (0.35, 1.97)	0.88 (0.34, 2.28)
		*P = 0.38*	*P = 0.67*	*P = 0.79*
**Pain located in the chest and abdomen and back**				
**Chest and abdomen pain**	**N (no/yes)**	**Unadjusted OR (95% CI) P value**	Multiply adjusted for confounders*	Additionally adjusted for other pain locations**
PTSD	22/32	1.00	1.00	1.00
	32/26	0.56 (0.26, 1.18)	0.60 (0.26, 1.36)	0.56 (0.23, 1.33)
		*P = 0.13*	*P = 0.22*	*P = 0.19*
Number of injuries	27/7	1.00	1.00	1.00
	34/55	6.24 (2.45, 15.89)	7.93 (2.58, 24.36)	8.18 (2.53, 26.41)
		*P < 0.001*	*P < 0.001*	*P < 0.001*
Anger in last 2 weeks	9/2	1.00	1.00	1.00
	52/60	5.19 (1.07, 25.12)	3.70 (0.60, 22.81)	2.67 (0.39, 18.45)
		*P < 0.05*	*P = 0.16*	*P = 0.32*
Aggressiveness in last 2 weeks	22/21	1.00	1.00	1.00
	39/41	1.10 (0.52, 2.31)	0.83 (0.34, 2.04)	0.76 (0.30, 1.97)
		*P = 0.80*	*P = 0.68*	*P = 0.58*
Crying in last 2 weeks	30/7	1.00	1.00	1.00
	33/55	7.12 (2.8, 18.12)	12.95 (3.87, 43.33)	13.15 (3.82-45.25)
		*P < 0.001*	*P < 0.001*	*P < 0.001*
Hatred in last 2 weeks	24/24	1.00	1.00	1.00
	37/38	1.03 (0.50, 2.12)	1.01 (0.44, 2.33)	0.83 (0.34, 2.02)
		*P = 0.94*	*P = 0.98*	*P = 0.69*
**Back pain**	**N (no/yes)**	**Unadjusted OR (95% CI) P value**	Multiply adjusted for confounders*	Additionally adjusted for other pain locations**
PTSD	28/26	1.00	1.00	1.00
	32/26	0.88 (0.42, 1.84)	0.86 (0.39, 1.89)	1.18 (0.50, 2.77)
		*P = 0.73*	*P = 0.71*	*P = 0.70*
Number of injuries	25/9	1.00	1.00	1.00
	45/44	2.72 (1.14, 6.47)	1.97 (0.76, 5.10)	1.37 (0.49, 3.87)
		*P < 0.05*	*P = 0.16*	*P = 0.55*
Anger in last 2 weeks	11/0	-	-	-
	59/53			
		*-*	*-*	*-*
Aggressiveness in last 2 weeks	28/15	1.00	1.00	1.00
	42/38	1.69 (0.79, 3.63)	1.24 (0.53, 2.93)	1.70 (0.67, 4.33)
		*P = 0.18*	*P = 0.62*	*P = 0.27*
Crying in last 2 weeks	25/12	1.00	1.00	1.00
	47/41	1.78 (0.79, 4.01)	2.00 (0.79, 4.99)	1.69 (0.60, 4.78)
		*P = 0.16*	*P = 0.14*	*P = 0.32*
Hatred in last 2 weeks	33/15	1.00	1.00	1.00
	37/38	2.26 (1.06, 4.83)	2.82 (1.20, 6.65)	3.53 (1.39, 8.97)
		*P < 0.05*	*P < 0.05*	*P < 0.01*
**Pain located in the chest and abdomen and back**				
**Upper limbs pain**	**N (no/yes)**	**Unadjusted OR (95% CI) P value**	Multiply adjusted for confounders*	Additionally adjusted for other pain locations**
PTSD	48/6	1.00	1.00	1.00
	47/11	1.87 (0.64, 5.47)	2.25 (0.66, 7.71)	2.87 (0.71, 11.51)
		*P = 0.25*	*P = 0.20*	*P = 0.14*
Number of injuries	28/6	1.00	1.00	1.00
	76/13	0.80 (0.28, 2.30)	1.07 (0.31, 3.74)	1.08 (0.26, 4.53)
		*P = 0.68*	*P = 0.91*	*P = 0.91*
Anger in last 2 weeks	11/0	-	-	-
	93/19			
		*-*	*-*	*-*
Aggressiveness in last 2 weeks	37/6	1.00	1.00	1.00
	67/13	1.20 (0.42, 3.41)	1.27 (0.37, 4.31)	1.22 (0.32, 4.60)
		*P = 0.74*	*P = 0.71*	*P = 0.77*
Crying in last 2 weeks	33/4	1.00	1.00	1.00
	75/13	1.41 (0.43, 4.64)	1.19 (0.29, 4.86)	0.63 (0.12, 3.30)
		*P = 0.58*	*P = 0.81*	*P = 0.59*
Hatred in last 2 weeks	41/7	1.00	1.00	1.00
	63/12	1.12 (0.41, 3.07)	0.71 (0.23, 2.19)	0.75 (0.22, 2.61)
		*P = 0.83*	*P = 0.55*	*P = 0.66*
**Lower limbs pain**	**N (no/yes)**	**Unadjusted OR (95% CI) P value**	Multiply adjusted for confounders*	Additionally adjusted for other pain locations**
PTSD	43/11	1.00	1.00	1.00
	40/18	1.76 (0.74, 4.18)	1.90 (0.76, 4.71)	2.11 (0.82, 5.45)
		*P = 0.20*	*P = 0.17*	*P = 0.12*
Number of injuries	25/9	1.00	1.00	1.00
	66/23	0.97 (0.39, 2.38)	0.82 (0.30, 2.27)	0.79 (0.26, 2.39)
		*P = 0.94*	*P = 0.70*	*P = 0.67*
Anger in last 2 weeks	11/0	-	-	-
	80/32			
		*-*	*-*	*-*
Aggressiveness in last 2 weeks	32/11	1.00	1.00	1.00
	59/21	1.04 (0.44, 2.42)	0.97 (0.37, 2.51)	1.19 (0.43, 3.30)
		*P = 0.94*	*P = 0.95*	*P = 0.74*
Crying in last 2 weeks	28/9	1.00	1.00	1.00
	65/32	1.08 (0.44, 2.63)	1.32 (0.49, 3.58)	1.62 (0.53, 5.02)
		*P = 0.87*	*P = 0.58*	*P = 0.40*
Hatred in last 2 weeks	37/11	1.00	1.00	1.00
	54/21	1.31 (0.56, 3.03)	1.36 (0.55, 3.38)	1.81 (0.68, 4.80)
		*0.53*	*P = 0.51*	*P = 0.23*

* Adjusted for sex, marital status, education, income, municipality and medication.

** Adjusted for sex, marital status, education, income, municipality, medication and additionally pain in chest and abdomen, back, upper and lower limbs.

The sensory pain score and PRI increased with a higher number of pain locations (Table 3). There was no association between the affective pain score and the number of pain locations. A higher sensory pain score was observed in participants with head, neck and shoulder or back pain, while a higher affective pain score was more common in participants with neck and shoulder, chest and abdomen, or back pain. The participants were more likely to report constant pain if they marked more pain locations on body diagram (OR = 1.27, 95% CI: 1.02-1.58, P < 0.05). And the pain was more likely to be constant in those with headache (OR = 2.89, 95% CI: 1.06-7.85, P < 0.05) or neck and shoulder pain (OR = 3.79, 95%CI = 1.23-11.67, P < 0.05), reciprocally adjusted for all other pain locations.

**Table 3:** Pain characteristic and pain rating index compared by location and number of pain locations

**Score of sensory component**				
	**N (no/yes)**	**Unadjusted OR (95% CI) P value**	Multiply adjusted for confounders*	Reciprocally adjusted**
Head	68/49	2.34 (0.33, 4.35)	2.67 (0.76, 4.58)	3.10 (1.21, 4.99)
		*P < 0.05*	*P < 0.01*	*P < 0.01*
Neck and shoulder	86/31	3.28 (1.06, 5.50)	2.87 (0.77, 4.96)	2.63 (0.56, 4.69)
		*P < 0.01*	*P < 0.01*	*P < 0.01*
Chest and abdomen	60/57	0.81 (−1.21, 2.84)	0.03 (−2.03, 2.09)	−0.49 (−2.45, 1.46)
		*P = 0.43*	*P = 0.98*	*P = 0.62*
Back	65/52	2.63 (0.65, 4.61)	1.75 (−0.18, 3.68)	2.54 (0.63, 4.45)
		*P < 0.01*	*P = 0.08*	*P < 0.01*
Upper limbs	99/18	0.77 (−2.03, 3.58)	1.31 (−1.46, 4.08)	0.74 (−1.91, 3.38)
		*P = 0.59*	*P = 0.35*	*P = 0.58*
Lower limbs	85/32	0.08 (−2.19, 2.36)	0.07 (−2.07, 2.22)	0.71 (−1.32, 2.74)
		*P = 0.94*	*P = 0.95*	*P = 0.49*
Number of pain locations	(increase of 1)	0.85 (0.39, 1.32)	0.85 (0.40, 1.30)	
		*P < 0.001*	*P < 0.001*	
**Score of affective component**				
	**N (no/yes)**	**Unadjusted OR (95% CI)**	Multiply adjusted for confounders*	Reciprocally adjusted**
Head	72/49	0.12 (−1.46, 1.70)	0.24 (−1.19, 1.66)	0.46 (−0.97, 1.89)
		*P = 0.88*	*P = 0.75*	*P = 0.52*
Neck and shoulder	88/33	1.60 (−0.12, 3.32)	1.29 (−0.25, 2.83)	1.46 (−0.11, 3.02)
		*P = 0.07*	*P = 0.10*	*P = 0.07*
Chest and abdomen	60/61	2.21 (0.71, 3.71)	1.74 (0.29, 3.20)	1.67 (0.20, 3.15)
		*P < 0.01*	*P < 0.05*	*P < 0.05*
Back	68/53	1.59 (0.05, 3.12)	0.72 (−0.69, 2.13)	0.54 (−0.90, 1.98)
		*P < 0.05*	*P = 0.31*	*P = 0.46*
Upper limbs	102/19	−0.98 (−3.11, 1.15)	−0.71 (−2.68, 1.27)	−1.25 (−3.24, 0.75)
		*P = 0.36*	*P = 0.48*	*P = 0.22*
Lower limbs	89/32	0.72 (−1.03, 2.48)	0.59 (−0.97, 2.15)	0.66 (−0.89, 2.21)
		*P = 0.42*	*P = 0.46*	*P = 0.40*
Number of pain locations	(increase of 1)	0.07 (−0.31, 0.44)	0.03 (−0.32, 0.37)	
		*P = 0.73*	*P = 0.88*	
**Pain rating index**				
	**N (no/yes)**	**Unadjusted OR (95% CI)**	Multiply adjusted for confounders*	Reciprocally adjusted**
Head	68/49	2.41 (−0.77, 5.60)	2.99 (0.13, 5.84)	3.63 (0.80, 6.46)
		*P = 0.14*	*P < 0.05*	*P < 0.01*
Neck and shoulder	86/31	4.94 (1.46, 8.41)	4.19 (1.10, 7.27)	4.09 (1.00, 7.18)
		*P < 0.01*	*P < 0.01*	*P < 0.01*
Chest and abdomen	60/57	3.15 (0.03, 6.27)	1.74 (−1.27, 4.75)	1.10 (−1.83, 4.02)
		*P < 0.05*	*P = 0.26*	*P = 0.46*
Back	65/52	4.30 (1.21, 7.40)	2.56 (−0.29, 5.40)	3.16 (0.30, 6.02)
		*P < 0.01*	*P = 0.08*	*P < 0.05*
Upper limbs	99/18	−0.38 (−4.78, 4.01)	0.46 (−3.63, 4.55)	−0.57 (−4.53, 3.39)
		*P = 0.86*	*P = 0.83*	*P = 0.78*
Lower limbs	85/32	0.78 (−2.87, 4.33)	0.72 (−2.43, 3.87)	1.42 (−1.62, 4.46)
		*P = 0.67*	*P = 0.65*	*P = 0.36*
Number of pain locations	(increase of 1)	0.92 (0.18, 1.67)	0.87 (0.19, 1.56)	
		*P < 0.05*	*P < 0.01*	

* Adjusted for sex, marital status, education, income, municipality and medication.

** Adjusted for sex, marital status, education, income, municipality, medication and reciprocally for all other pain locations.

### Effects of mental and emotional disturbances and physical problems

The risks of having changed or stopped jobs or stopped schooling due to depression or injury were higher for those who reported most pain, and it was more significant in those with greater sensory dimensional pain (Table 4). After adjusting for the confounders, the risks also increased if the pain was in neck and shoulder (OR = 3.70, 95% CI: 1.41-9.73, P < 0.01, and OR = 2.96, 95% CI: 1.08-8.13, P < 0.05, additionally adjusted for other pain locations) and in the upper limbs (OR = 4.87, 95% CI: 1.33-17.84, P < 0.05, and OR = 3.98, 95% CI: 0.97-16.35, P = 0.06, additionally adjusted for other pain locations). The risk was higher in those with PTSD but the association was not significant (OR = 1.29, 95% CI = 0.48-3.46, P = 0.61). Associations with pain scores were stronger in terms of statistical significance but this may reflect greater statistical power associated with using continuous variable rather than greater importance.

**Table 4:** Odds ratios of career change due to depression or injury by pain characteristics and frequency

**Had changed jobs or stopped job or stopped schooling due to depression or injury**			
	**N (no/yes)**	**Unadjusted OR (95% CI) P value**	Multiply adjusted for confounders*
Pain frequency in last 2 weeks			
Periodic and occasional	46/12	1.00	1.00
Consistent	34/15	1.69 (0.70, 4.07)	1.74 (0.60, 5.01)
		*P = 0.24*	*P = 0.31*
Sensory component of pain**	92/29	2.00 (1.20, 3.33)	2.27 (1.17, 4.42)
		*P < 0.01*	*P < 0.05*
Affective component of pain**	89/28	1.70 (1.05, 2.75)	1.84 (1.05, 3.23)
		*P < 0.05*	*P < 0.05*
Pain rating index**	89/28	2.06 (1.21, 3.49)	2.44 (1.25, 4.76)
		*P < 0.01*	*P < 0.01*

* Adjusted for sex, marital status, education, income, municipality and medication.

**Associations with pain scores are presented as the change in odds associated with a 1 SD change in pain score to allow comparison between pain scores if needed.

Reporting of sleep disorders was substantially greater in those with higher number of pain locations and pain scores, in particular for score of sensory pain (OR = 3.99, 95% CI: 1.84-8.63, P < 0.001). Frequency of pain had little impact on sleep disorders after model adjustment. Association between sleep disorder and PTSD was not conventionally significant before and after the model adjustment. However, associations were found between various emotional disturbances and the reporting of sleep disorders. Anger, inferiority complex and military or police phobia were the strongest predictors for reporting of sleep disorders (Table 5), although the latter effect was attenuated by model adjustment.

**Table 5:** Odd ratios of sleep disorder and suicide ideation by mental, physical and emotional problems

	**Sleep disorder**			**Suicide ideation**		
	**N (no/yes)**	**Unadjusted OR (95% CI) P value**	Multiply adjusted for confounders*	**N (no/yes)**	**Unadjusted OR (95% CI) P value**	Multiply adjusted for confounders*
PTSD	10/44	1.00	1.00	16/38	1.00	1.00
	7/51	1.66 (0.58, 4.72)	1.83 (0.56, 6.04)	9/49	2.29 (0.91, 5.75)	3.61 (1.20, 10.86)
		*P = 0.35*	*P = 0.32*		*P = 0.08*	*0.02*
Number of injuries: 0	10/24	1.00	1.00	13/21	1.00	1.00
Number of injuries: ≥1	14/75	2.23 (0.88, 5.67)	1.46 (0.42, 5.08)	18/71	2.44 (1.03, 5.79)	2.23 (0.71, 6.97)
		*P = 0.09*	*P = 0.55*		*P < 0.05*	*0.17*
Pain frequency in last 2 weeks						
Periodic and occasional	13/45	1.00	1.00	18/40	1.00	1.00
Consistent	7/42	1.73 (0.63, 4.76)	0.90 (0.27, 3.07)	9/40	2.00 (0.80, 4.98)	1.67 (0.55, 5.05)
		*P = 0.29*	*P = 0.87*		*P = 0.14*	*P = 0.36*
Sensory component of pain	23/98	4.03 (2.20, 7.39)	3.52 (1.76, 7.03)	29/88	1.68 (1.11, 2.54)	1.68 (0.95, 2.96)
		*P < 0.001*	*P < 0.001*		*P < 0.05*	*P = 0.07*
Affective component of pain	21/96	2.35 (1.40, 3.95)	2.16 (1.14, 4.10)		1.70 (1.10, 2.64)	1.74 (0.99, 3.04)
		*P < 0.001*	*P < 0.05*	*31/90*	*P < 0.05*	*P < 0.05*
Pain rating index	21/96	3.74 (2.03, 6.90)	3.52 (1.71, 7.23)		1.86 (1.20, 2.89)	2.01 (1.09, 3.70)
		*P < 0.001*	*P < 0.001*	*29/88*	*P < 0.01*	*P < 0.05*
**Emotional disturbance**	**N (no/yes)**	**Unadjusted OR (95% CI) P value**	Multiply adjusted for confounders*	**N (no/yes)**	**Unadjusted OR (95% CI) P value**	Multiply adjusted for confounders*
Anger	8/3	1.00	1.00	6/5	1.00	1.00
	16/96	16 (3.83, 66.76)	24.62 (3.30, 183.49)	25/87	4.18 (1.18, 14.83)	2.69 (0.52, 13.93)
		*P < 0.001*	*P < 0.01*		*P < 0.05*	*P = 0.24*
Aggressiveness	16/27	1.00	1.00	15/28	1.00	1.00
	8/72	5.33 (2.05, 13.89)	4.11 (1.29-13.07)	16/64	2.14 (0.93, 4.93)	1.47 (0.52, 4.18)
		*P < 0.001*	*P < 0.05*		*P = 0.07*	*P = 0.47*
Crying	13/23	1.00	1.00	17/19	1.00	1.00
	11/76	3.90 (1.54, 9.88)	4.26 (1.20, 15.09)	14/73	4.67 (1.96, 11.12)	4.37 (1.46, 13.04)
		*P < 0.01*	*P < 0.05*		*P = 0.40*	*P = 0.30*
Hatred	13/35	1.00	1.00	20/28	1.00	1.00
	11/64	2.16 (0.88, 5.33)	1.88 (0.62, 5.70)	11/64	4.16 (1.76, 9.81)	2.59 (0.99, 6.79)
		*P = 0.09*	*P = 0.267*		*P < 0.001*	*P < 0.05*
Helplessness	12/23	1.00	1.00	15/20	1.00	1.00
	12/76	3.30 (1.31, 8.34)	5.28 (1.51, 18.40)	16/72	3.38 (1.43, 7.99)	2.48 (0.92, 6.67)
		*P < 0.01*	*P < 0.01*		*P < 0.01*	*P = 0.07*
Hopeless	15/24	1.00	1.00	13/26	1.00	1.00
	9/75	5.21 (2.03, 13.41)	4.45 (1.41, 14.04)	18/66	1.83 (0.79, 4.27)	0.92 (0.33, 2.58)
		*P < 0.001*	*P < 0.01*		*P = 0.16*	*P = 0.88*
Inferiority complex	17/26	1.00	1.00	17/26	1.00	1.00
	7/73	6.82 (2.54, 18.30)	5.96 (1.81, 19.60)	14/66	3.08 (1.33, 7.14)	2.67 (0.98, 7.31)
		*P < 0.001*	*P < 0.01*		*P < 0.01*	*P = 0.06*
Military or police phobia	17/21	1.00	1.00	15/23	1.00	1.00
	7/78	9.02 (3.31, 24.60)	4.53 (1.40, 14.64)	16/69	2.81 (1.20, 6.57)	1.62 (0.56, 4.68)
		*P < 0.001*	*P < 0.01*		*P < 0.05*	*P = 0.37*
Sadness	13/38	1.00	1.00	18/33	1.00	1.00
	11/61	1.90 (0.77, 4.66)	2.31 (0.74, 7.23)	13/59	2.48 (1.08, 5.68)	2.09 (0.77, 5.73)
		*P = 0.16*	*P = 0.15*		*P < 0.05*	*P = 0.15*

* Adjusted for sex, marital status, education, income, municipality and medication.

PTSD was a strong predictor for suicide ideation (OR = 3.61, 95% CI = 1.20-10.86, P < 0.05) (Table 5). Emotional disturbance such as anger, recent crying and hatred were the most marked disturbance expressed by those with suicide ideation (Table 5). However, the latter effect was attenuated by adjustment in the model. Suicide ideation also increased with at least one injury, the number of pain locations (OR = 1.51, 95% CI: 1.09-2.10, P < 0.01) and increasing pain scores regardless of the measurement of pain used (ORs per SD increase were very similar for the scores of sensory and affective dimensional pain and PRI).

## Discussion

The study collected information about the history of trauma among 125 victims in Mitrovicë district who had been exposed to torture or massive violence. Around 40% of them were affiliated with KLA and 20% had been in a combat or cross-fire situation. We did not obtain further information to define their affiliation with KLA, therefore we don’t know whether they were the combatants, informers or civilians who helped KLA members, etc. Civilians could be caught in the cross-fire situation too. Self-selection bias in the sample may exist although we do not know what proportion of population was affiliated with the KLA during the wartime.

One of the most frequent symptoms among the study population was pain; this was often present together with PTSD. The situation is not clear-cut – a lot of symptoms are frequent among those who do not have PTSD diagnosis. Our results showed that there is often a substantial overlap between pain, PTSD and emotional disturbance (such as frequent anger or feelings of hatred and aggressiveness). Many studies have also shown comorbidity of PTSD and somatic symptoms such as pain. One of explanations that has been given for this comorbidity is that pain and PTSD emerge from shared biological diatheses [23].

Pain is often regarded as secondary symptom to PTSD rather than a separate response to trauma. PTSD has been considered to be an affective disorder, but our results showed that the participants with pain and PTSD tended to have the same score for the affective dimension of pain as the participants without PTSD. On the other hand, there were differences; those with PTSD had a higher score for sensory dimensional pain than those without PTSD. We do not have any neurobiological explanation of this phenomenon from our study, but the literature does suggest that there may be neurobiological consequences of traumatic stress, and comorbid mental problems [24,25]. In the present study, in which physical and injury examination conducted by medical doctors was compared with participants’ oral reports, the overall pain rating was strongly associated with having at least one injury, whereas the association with PTSD was weaker. In our previous publication, we reported the most frequent bodily locations of injuries, which also correspond to the most frequent bodily location of pain complaints in this paper. This indicates that at least some of pain complaints in this study could be simply attributed to the actual injuries suffered. The problems may have been exacerbated by the sensitization of peripheral receptors following injury, which can be responsible for post-injury pain hypersensitivity [26]. Mental and physical residual effect to torture and massive violence-related trauma are complicated and vary widely according to an individual’s physical condition and coping skill as well as environmental factors like the kind of support available. It is difficult to say how many of the pain complaints in our study participants were secondary symptoms in response to PTSD and how many were direct and separate responses to physical damage or poor bodily functioning due to torture and physical punishment.

However, we did find indications that factors other than physical injury do play a role, because particular pain locations were also related to various aspects of negative emotional processing. Previous studies have shown similar connections [27-29]. Anger was the strongest predictor of pain in our sample, and anger-induced pain occurred more persistently in head, chest and abdomen. Our study also found an association that has not been previously described, between crying and chest and abdominal pain experience within 2 weeks preceding the survey, which was associated with greater score for affective dimensional pain. However, pain after crying was found to be associated with injury, as 42% of participants had injuries of the chest due to torture or other types of physical punishment. It is possible that anger and crying make certain muscles so tense that this causes pain due to previous physical damage which may not have been treated adequately. But it is also likely that the experience of negative emotion and desire for relief could influence the pain effect, and pain-related emotions can influence pain perception and pain-related physiological response [30].

In all discussions of symptoms related to trauma it must be borne in mind that cross-cultural studies have shown that these symptoms are experienced and expressed in different ways by people from different cultures and different genders. For example, in some cultural settings pain complaints can be a verbal expression of depression. This could lead to misleading results [31-33]. However, one strength of our study is that the physical examinations and clinical diagnoses of PTSD were carried out on the basis of DSM-IV criteria by three experienced medical doctors who come from the same ethnic and cultural background as the study participants.

One aspect of our study was a consideration of how far anger and hatred influenced the experience of pain and PTSD among victims. Victims of torture and massive violence in Kosovo do not only suffer from the mental and physical effects of their experience, but feel strongly that they have been treated unjustly. More than half of participants experienced hatred and a desire for revenge – even though one fifth of them stated that the perpetrators had been prosecuted. There are some encouraging trends; for example it has been shown that in 2000, 54% of men of Albanian ethnicity in Kosovo felt hatred toward the Serbs, compared with 88.7% in 1999 [18,19]. However, figures for the general population hide the fact that there may be many people in post-war Kosovo who, like the participants in our study, are still filled with anger, hatred and a desire for revenge. It seems that many victims have found no major emotional outlet enabling them to handle unbearable suffering. In some cultural contexts, repressed anger turns inward, resulting in self-harm. In other cultural contexts, depression or feelings of inferiority turn outwards and are converted into rage or hatred which often erupts in violence and leads to destruction. Such emotional hurt can be very contagious for family members and friends. It has been observed in Kosovo and other similar settings in the Balkans that there has been a rapid growth of consumption of analgesics and antidepressants and that the use of analgesics and the frequency of medical visits were significantly higher in the presence of PTSD and major depression [34]. Half of our study participants were taking medications against depression and anxiety during the time of the survey, but these seemed to have failed to relieve their symptoms. It is well-known that psychiatric symptoms affect the effectiveness of treatment for pain [6,35] and that chronic pain is often refractory to treatment [36]. Health professionals need to bear in mind that the presence of negative emotions such as anger and hatred may limit the efficacy of medications for PTSD. These emotions may also reduce the efficacy of pain management, which is important as pain is often inadequately treated and can become one of the most costly and disabling conditions. More clinical attention should be given to anger-hatred management in individuals with pain [29]. International guidelines for treatment of torture victims suggest that addressing emotional factors would improve the treatment outcome in many cases [37-39]. Interventions specifically focused on depression and pain management using medication regulating the mood and the perception of pain alone might well fail in victims of torture and massive violence; therefore a stress management approach that includes relaxation training, cognitive restructuring, and problem-solving skills combined with physiotherapy or art therapy are recommended to improve the residual symptoms of PTSD [40-42].

Pain management is of vital importance, whether the cause is a result of physical injury or is at least partly emotional. Patients with comorbidity of mental disorders and painful physical symptoms show more emotional distress, poorer physical functioning and lower rates of help seeking [7]. They also have worse prospects in the labor market and more financial problems and disability [43]. Overall pain scores were higher in those who had changed jobs or stopped doing jobs or going to school due to depression or injury. These risks were higher in the presence of neck and shoulder or upper limb pain, which can lead to consequences that make employment and coping with practical activities especially difficult. The results published earlier showed that there were more than 50% who had been injured on their head, 20% on neck or shoulder and 15% on upper back [15]. Shoulder and back muscles are often the first body location affected by tension. This may lead to more pain in the neck and shoulders or upper limbs. This could be relevant to our earlier observation that, among female participants, having more emotional disturbances affected the handgrip strength – which is needed for many types of work.

Persistent difficulty falling or staying asleep is one of clinical criteria for PTSD, but it also occurred in 41 participants (32%) in the study who had not been diagnosed with PTSD. It is possible that some of sleep disturbance was attributable to present life stressors. Besides confirming the association between pain and negative mood and poor sleep quality [44], our study also indicated for the first time an association between anger, military or police phobia and self-reported sleep disorder. Since the Serbian police and paramilitary were responsible for most of the attacks at their homes and on the streets, many of the victims had reported police or military phobia. Our result is in line with the literature suggesting that sleep disorders are associated with war-related exposure [45-48]. Previous study demonstrated that awaking thresholds depend on the severity of depression and anxiety in war-related PTSD patients [49]. Therefore, we recommend integrating “Exposure Therapy”, which has been proven to be effective in the treatment of PTSD, anxiety disorder and specific phobia, in the rehabilitation intervention [50-54].

Suicide ideation was extremely high and was related to PTSD and emotional disturbances such as feelings of anger, hatred and recent crying, as well as higher pain score. Recently, Wenzel T *et al.*[17] reported that suicide ideation was related both to past stressful experience (depression) and present social stressors (unemployment) among the general population in Kosovo. Many participants in our study were not only suffering from trauma in the past but also had no jobs and felt hopeless at the time of the study, so these could be the factors contributing to the recurrent suicide thoughts.

It will be of great benefit if research can refine current understanding of the interaction between pain and emotional processing in the brain and its connection with other physical and psychological variables after trauma. Understanding the inter-relationships among injury history, location of pain, reduction of muscle strength and other functional disability, poor career outcomes and their impact on the post-war economy will be crucial for developmental work.

There will be a need to continue with health interventions in the Balkan region. Many problems need to be addressed, ranging from heavy consumption of analgesics and antidepressants, sleep impairment, unemployment to suicide rate. As our study shows, the situation is frequently complicated by comorbidity of mental and/or physical problems. It is evident that treatments need to address cognitive and emotional integration and the residual effect of injury. Further research is also warranted to define appropriate outcome measures, and evaluate the effect of different intervention modalities which aim to reduce the psychopathology and physical and emotional suffering in the post-war setting [42,53-55].

Many of our study participants and their relatives were affiliated with Kosovo Liberation Army (KLA). Some KLA members who were once freedom fighters now enter into the law enforcement agencies and politics in Kosovo. Collective emotions play a critical role in shaping individual and societal responses to conflicting events and ultimately influence the success or failure of resolution and post-conflict reconciliation attempts [56,57]. Bar-Tal D further suggest that up-regulating hope and down-regulating hatred can have constructive implications in the post-reconciliation stage of intragroup conflict [56]. It is very unfortunate that provocative actions of authorities in both Pristina and Belgrade have lead to re-experiencing of trauma and violent clashes in Mitrovicë in the post-war era.

### Limitations

Current findings point to several issues that must be addressed in future research. One is the question of how representative a sample can be in this setting. The study participants were a self-selected sample among the victims of torture and massive violence identified in a household survey. There were many others who fulfilled the same criteria, but chose not to come to the clinic for further examinations. A second source of bias which is inevitable in a post-conflict setting is that a study sample can only include people who have survived, and have stayed in Kosovo. We found few people who had undergone torture or abuse during the first decade of Slobodan Milošević’s administration until 1996. This was most likely because many of those most terrified had gone into exile and settled down abroad. Survival bias inevitably exists in a cross-sectional study in a post-conflict setting.

Besides the inevitable bias in sample selection, there may have been bias among physicians arising from the fact that we used a clinical diagnosis of PTSD as the “gold standard”, and did not investigate PTSD in a dimensional perspective using a standard questionnaire. The study is also limited by the fact that for military or police phobia, sleep disorder as well as suicide ideation we had to rely on self-reporting. The assessment of pain was important for the current study. The standard McGill Pain Questionnaire (MPQ) has been proven to be able to assess different dimensions of pain in many different cultural settings [13]. Although cross-cultural adaptation was taken into account, clinimetric testing was not undertaken in Kosovo. Therefore the interpretation of the results focused on the overall pain rating index rather than the affective and sensory dimensions of pain.

## Conclusions

We provided a general overview of comorbid pain and PTSD in 125 victims of torture and massive violence in Kosovo, and further established the association of emotional disturbance such as feeling anger and hatred with pain locations and PTSD. MPQ is a simple, inexpensive and indicative tool for pain assessment; however, the Albanian versions of the questionnaires need to be validated for widespread use. In spite of some limitations, the study adds to a growing literature addressing pain modalities and the independent and interactive effects of pain and PTSD. The findings have implications for various treatment modalities of victims of torture and massive violence and suggest new approaches to rehabilitation and peace-building in a post-war setting.

## Abbreviations

DSM-IV: American Psychiatric Association’s Diagnostic and Statistical Manual of Mental Disorders; CI: Confidence interval; KLA: Kosovo Liberation Army; KRCT: Kosovo Rehabilitation Centre for Torture Victims; MPQ: McGill Pain Questionnaire; NATO: North Atlantic Treaty Organization; NGO: Non-governmental organisation; OR: Odds ratio; PRI: Pain Rating Index; PTSD: Post-traumatic stress disorder; RCT: Rehabilitation and Research Centre for Torture Victims; SD: Standard deviation.

## Competing interests

The authors declare that they have no competing interests. There is no relationship between authors and sponsors which could potentially bias the results.

## Authors’ contributions

SW participated in the design of the study, conducted the field work, analysed and interpreted data and drafted the manuscript. Three medical doctors: FR, XS, SP carried out the clinical and functional assessments. FR, FS, and BG assisted in data collection, coordination and supervision. JMO participated in the conception of the work, helped to draft the manuscript and revised it critically at all stages. All authors read and approved the final manuscript.
